# Intraoperative Radiation Therapy: A Critical Analysis of the ELIOT and TARGIT Trials. Part 2—TARGIT

**DOI:** 10.1245/s10434-014-3999-5

**Published:** 2014-08-20

**Authors:** Melvin J. Silverstein, Gerd Fastner, Sergio Maluta, Roland Reitsamer, Donald A. Goer, Frank Vicini, David Wazer

**Affiliations:** 1 Breast Center, Hoag Memorial Hospital Presbyterian, Newport Beach, CA USA; 2 Department of Surgery, Keck School of Medicine, University of Southern California, Los Angeles, CA USA; 3 Radiotherapy and Radio-Oncology Landeskrankenhaus, Paracelsus Medical University, Salzburg, Austria; 4 Radiation Oncology, Oncological Hyperthermia Unit—Medical Center, Verona, Italy; 5 Department of Senology, Paracelsus Medical University Salzburg, Salzburg, Austria; 6 Physics, IntraOp Medical, Sunnyvale, CA USA; 7 Radiation Oncology, St. Joseph Mercy Oakland, Pontiac, MI USA; 8 Radiation Oncology, Tufts University School of Medicine, Boston, MA USA; 9 Radiation Oncology, Albert Medical School of Brown University, Providence, RI USA

## Abstract

**Background:**

Two randomized intraoperative radiation therapy trials for early-stage breast cancer were recently published. The ELIOT Trial used electrons (IOERT), and the TARGIT-A Trial Update used 50-kV X-rays (IORT). These studies were compared for similarities and differences. The results were analyzed and used to determine which patients might be suitable for single-dose treatment.

**Methods:**

The primary sources of data were the ELIOT Trial and TARGIT-A Trial, as well as a comprehensive analysis of the peer-reviewed literature of accelerated partial breast irradiation (APBI) using 50-kV X-rays or electrons. Studies published or presented prior to March 2014 were analyzed for efficacy, patient restrictions, complications, and outcome.

**Results:**

With a median follow-up of 5.8 years, the 5-year recurrence rates for ELIOT versus EBRT patients were 4.4 and 0.4 %, respectively, *p* = 0.0001. A low-risk ELIOT group was identified with a 5-year recurrence rate of 1.5 %. With a median follow-up of 29 months, the 5-year recurrence rates for the TARGIT-A versus EBRT patients were 3.3 and 1.3 %, respectively, *p* = 0.042.

**Conclusions:**

With 5.8 years of median follow-up, IOERT appears to have a subset of low risk women for whom IOERT is acceptable. With 29 months of median follow-up the results of IORT with 50-kV devices are promising, but longer follow-up data are required. At the current time, single-fraction IOERT or IORT patients should be treated under strict institutional protocols.

In the preceding report (Part 1) in this issue of the *Annals*, we outline the rationale for intraoperative radiation therapy (IORT) and begin a critical analysis of the 2 prospective randomized trials currently published. Part 1 discusses the ELIOT Trial, a trial using electrons during surgery as the entire radiation therapy treatment. In this report, we continue with a critical analysis of the TARGIT-A Trial, a trial that used 50-kV x-rays rather than electrons.

## METHODS

See Part I for methods used in the analysis.

### TARGIT-A Trial

#### Overview

The TARGIT-A Trial randomized 3,451 patients either to standard EBRT treatment or to TARGIT-A. Eligibility criteria were age ≥ 45 years, tumor size ≤ 3.5 cm, N0–1, M0, and unifocal invasive ductal carcinoma. If the participating institution determined the patient was at high risk for recurrence, an additional 5 weeks of EBRT was given, calling this “risk-adapted IORT.” The Trial began in March 2000. Beginning in 2004, approximately 30 % of the patients had TARGIT-A after final pathology in a second surgical procedure about 30 days after the original surgery. This group was designated the “postpathology” group as opposed to the “prepathology” group who received TARGIT-A during initial tumor surgery. The results for these different patient cohorts are shown in Table 1.

**Table 1:** TARGIT-A Trial results as reported over time

STUDY	Median Follow-up	Local recurrences					Any breast event				
		Targit	EBRT	Total	% Diff.	*p* value	Targit	EBRT	% Diff.	*p* value	
Lancet 2010	25 months	6	5	11	0.25	NS	10	8	NS	NS	
SABCS 2011	32 months	NS	NS	23	NS	NS	Not Stated				
SABCS 2012	29 months	23	11	34	2.01	0.042	69	48	2.5	0.053	

Lancet 2013	29 months	Local recurrences: total cohort					Any other breast event: total cohort				
All recurrence rates are 5-year Kaplan–Meir projections.		Targit	%	EBRT	%	*p* value	Targit	%	EBRT	%	*p* value
		23	3.3	11	1.3	0.042	46	4.9	37	4.4	NS
		Local recurrences: pre-pathology group					Any other breast event: pre-pathology group				
		Targit	%	EBRT	%	*p* value	Targit	%	EBRT	%	*p* value
		10	2.1	6	1.1	0.31	29	4.8	25	4.7	0.72
		Local recurrences: post-pathology group					Any other breast event: post-pathology group				
		Targit	%	EBRT	%	*p* value	Targit	%	EBRT	%	*p* value
		13	5.4	5	1.7	0.069	17	5.2	12	3.7	NS

Loco-regional Recurrences^a^ (*p* values NS in Lancet 2013)	Total target	Total EBRT	Targit pre-pathology	EBRT pre-pathology	Targit post-pathology	EBRT post-pathology
	4.2% *N* = 31	2.0 % *N* = 17	3.1 %	2.0 %	6.2 %	2.0 %
Regional recurrences	1.1 %	0.9%				
Distant recurrences	3.9 %	3.2%				
All recurrences^b^ (*p* values NS in Lancet 2013	8.2 % 69	5.7 % 48	6.9 % 39	5.8 % 31	10.4 % 30	5.4 % 17

Lancet 2013-Appendix	Pre-pathology targit only (*N* = 793)	Pre-pathology Targit + EBRT (*N* = 219)	Post-pathology targit only (*N* = 539)
5-year projected recurrence	2.7 %	0.9 %	5.9 %

Adapted and reprinted with permission of Springer Science & Business Media^24^

*NS* not stated

Lancet 2010: No metastatic events reported. 7 LRR reported. SABCS 2011 Poster: Results kept blinded. Only total number of local recurrences reported. SABCS 2012 Presentation: Targit recurrence was 3.3 %, HR = 2.05 (1.01–4.25). Lancet 2013: Distant recurrences and regional recurrences not reported separately

Total Targit plus EBRT metastases = 62 from SABCS 2012 Presentation

^a^ *p* values NS in Lancet 2013, but given as .02, HR = 2.2 (1.2–4.2) at SABCS 2012 for total cohort

^b^ *p* values NS in Lancet 2013, but given as .053, HR = 1.44 (.99–2.08) at SABCS 2012 for total cohort

#### Technique

In the prepathology TARGIT-A patients, following tumor excision, an appropriately sized spherical applicator was placed in the tumor bed. Purse string sutures were used to approximate breast tissue at risk to the applicator. Radiation was delivered over 20–45 min to the tumor bed, which received 20 Gy at the surface of the applicator and attenuated to 5–7 Gy at 1-cm depth. If risk factors were found at the time of surgery or postoperatively, when final pathology was available, the 20 Gy TARGIT treatment was considered as a boost, and patients received an additional 50 Gy equivalent of EBRT, delivered over 3–5 weeks, depending on the institutional preference. Institutions were free to determine what risk factors required additional EBRT.

The postpathology TARGIT-A patients received 20 Gy irradiation after final pathology determined no risk factors, typically within 30 days of surgical tumor removal.

The EBRT patients, whether prepathology or postpathology, received 3–5 weeks of 50 Gy equivalent EBRT ± boost depending on the institutional preference.

#### Complications

Wound complications were similar between groups, but grade 3 or 4 skin complications were significantly reduced with TARGIT (4 of 1720) vs EBRT (13 of 1731), *p* = 0.029.

#### Regional Failures

Regional failures were similar in both groups (8 events for TARGIT vs 6 events for EBRT) (*p* = 0.6).

#### Results

At 29 months of median follow-up, the 5-year risk of local recurrence was 1.3 % for EBRT and 3.3 % for all TARGIT-A patients (*p* = 0.042). Target A prepathology patients had a 5-year risk of 2.1 %. Postpathology patients had a 5-year risk of 5.7 %.

**Fig. 1:**
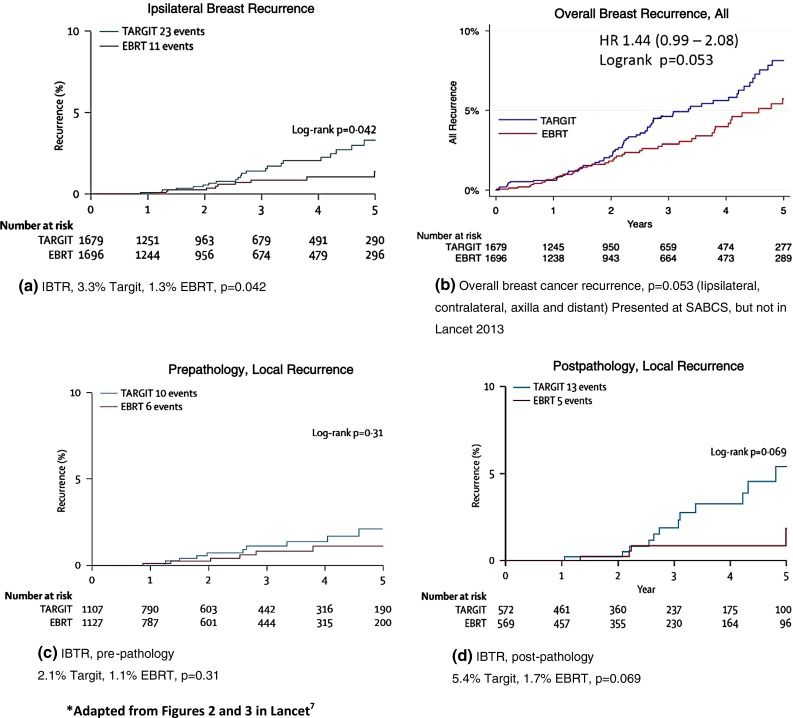
5-year Kaplan–Meier projections for recurrences from TARGIT-A treated patients vs EBRT treated patients. **a** Ipsilateral breast recurrence. **b** Overall breast recurrence. **c** Prepathology, local recurrence. **d** Postpathology, local recurrence. Adapted from Figs. 2 and 3 in Lancet^7^

Overall recurrence (ipsilateral breast, contralateral breast, axilla, and distant) showed a worsening trend for TARGIT A compared with EBRT: 69 events vs 48 events (*p* = 0.053). Both postpathology and prepathology TARGIT-A patients had more local recurrences than the EBRT patients, although the difference was not statistically significant. Postpathology patients exceeded the Trial’s preset noninferiority margin of 2.5 % (5.4 vs 1.7 %, *p* = 0.069); prepathology patients did not (2.1 vs 1.0 %, *p* = 0.31). Approximately 21 % of prepathology patients who received TARGIT-A also had 5 weeks of EBRT because of risk factors determined at the time of surgery or when final histopathology was available. Patients who received only TARGIT-A had 3 times the recurrence rate of those who received TARGIT-A plus 5 weeks of EBRT (2.7 vs 0.9 %). This difference was not significant, but no *p* value was provided. Ipsilateral breast recurrence rates for all patients, for prepathology and postpathology patients, and for any breast recurrence are shown in Fig. 1.

#### Survival

Breast cancer mortality was similar for TARGIT (2.6 %) vs EBRT (1.9 %), *p* = 0.56. TARGIT resulted in significantly fewer non-breast-cancer deaths 1.4 % (*n* = 17) vs 3.5 % (*n* = 35), *p* = 0.0086. This was due to fewer deaths from cardiovascular causes and other cancers. Overall mortality was 3.9 % for TARGIT versus 5.3 % for EBRT, *p* = 0.099.

## Discussion

Between March 2000 and April 2010 2,232 patients were accrued, sufficient for proof of noninferiority.^1^ Results were reported 3 months after completion of accrual when the median follow-up was 25 months.^1^ The authors maintained early publication was justified because proof of noninferiority required only 585 patients, and they had reached that number with a 4.6-year median follow-up. Also they said peak recurrences for breast cancer occur in years 2 and 3, offering in support that no recurrences were seen in year 4. At that time, critics expressed concern mainly about the immaturity of the data.^2^^–^ 6 Accrual and randomization of 1,219 additional patients continued until June 2012, increasing the Trial population to 3,451 patients, resulting in a median follow-up of just 29 months.^7^

The TARGIT-A update shows recurrences in both the TARGIT and EBRT groups in year 4.

At the time of the update, the 5 EBRT recurrences initially reported more than doubled to 11, and the six initial TARGIT recurrences had almost quadrupled to 23, questioning the claim of a recurrence peak at 2 or 3 years.^1^^,^ 7 The results of the TARGIT-A trial, with a median follow-up (FU) of 29 months, is still well below the median time when breast recurrences can be expected, especially since more than 90 % of TARGIT-A women were estrogen receptor positive, and at least 65 % received adjuvant hormonal therapy, a treatment well-known to delay recurrences in ER + women.^1^^,^ 7 ^–^ 9

The authors used binomial proportion statistics to show equivalence between the mature cohort (2,232 patients, median FU = 3 years, 7 months), the earliest cohort (1,222 patients, median FU = 5 years), and the total cohort (3,451 patients, median FU = 2 years 5 months). Haviland points out that binomial proportion statistics is invalid for follow-ups less than 5 years and that the appropriate statistical methodology is survival analysis for local recurrence.^10^ Only 18 % of patients had a FU of 5 years in the TARGIT-A update.^7^ Haviland estimates the hazard ratio for the reported local recurrence rates and calculates the local recurrence rate for TARGIT-A could be as high as 7.1 %, far exceeding the noninferiority margin of 2.5 % established by the trial.

The initial TARGIT-A publication did not differentiate between prepathology and postpathology patients or Targit boost patients.^1^ The TARGIT update shows these strata are not equivalent, with postpathology having higher local recurrence rates than prepathology (Table 2), despite postpathology patients presumably being lower risk as the treatment was delivered in a second operation after final pathology.^7^ The authors attribute the difference either to delay in wound fluid suppression of tumor cells, since there is a delay of radiation in postpathology TARGIT, or to a geometric miss when inserting the applicator postsurgery. While geometric miss might partially explain the results, it is not the likely a major cause of their findings. The IORT Intrabeam boost study of 299 patients reported no difference in recurrence rates between prepathology and postpathology patients.^11^ The 5-year recurrence rate for all patients was 1.73 %. The authors do not report the median applicator size used in the prepathology and postpathology patients, but if the median sizes reported in other Intrabeam publications are used, it is likely that postpathology patients had irradiated tissue volumes less than half the volumes in prepathology patients.^11^^,^ 12 In IORT boost, EBRT can compensate for the smaller volume irradiated in the postpathology patients. One can also see this trend in the prepathology TARGIT patients since TARGIT plus EBRT has three times fewer local recurrences than TARGIT alone even though those who also received 5 weeks of EBRT were presumably at higher risk (Table 2).

**Table 2:** TARGIT-A local recurrence summary by treatment cohort

Cohort	Number of recurrences	Percent	*p* value
Prepathology Targit	10	2.1 %	0.31
Prepathology EBRT	6	1.1 %	
Postpathology Targit	13	5.4 %	0.069
Postpathology EBRT	5	1.7 %	
Prepathology Targit alone (*N* = 793)	~7^a^	2.7 %	Not stated
Prepathology Targit + boost (*N* = 219)	~3^a^	0.9 %	

Reprinted with permission of Springer Science & Business Media^24^

^a^Number of recurrences extrapolated from presented data

The authors note that the difference in IBTR for all patients is still within their absolute noninferiority margin of 2.5 % (Fig. 1a).^7^ Cuzick cautions that the authors have misused the noninferiority criterion, which requires the upper confidence interval (CI) be less than the predefined noninferiority level of 2.5 %.^13^ In the TARGIT-A update, the upper CI was 5.1 %, throwing doubt on their assertion of noninferiority.^7^ Looking at the divergence of slopes in Fig. 1a, it appears likely that the 2.5 % noninferiority criterion for IBTR will be exceeded irrespective of the CI upper limit.

Overall breast recurrence rates in the TARGIT group also exceeded rates in the EBRT group (Fig. 1b), a difference at borderline statistical significance (*p* = 0.053).^14^ While the difference in breast cancer deaths with TARGIT vs EBRT is not significant (20 deaths, 2.6 % vs. 16 deaths, 1.9 %, *p* = 0.56), these higher recurrence rates with short follow-up suggests more follow-up is needed.

Follow-up may also be too short to determine whether prepathology TARGIT patients will ultimately do better than the entire TARGIT cohort. The difference between this favorable TARGIT cohort and the EBRT group is 1.0 %, with a median follow-up of 29 months, compared with a difference of .25 % between the TARGIT group and the EBRT group in the initial publication.^1^^,^ 7

The TARGIT study involved 33 centers in 11 countries and lasted more than 12 years. A large multi-institutional study such as TARGIT-A demands a high level of control and standardization. However, in TARGIT-A, each center treated the EBRT group according to its own institutional guidelines and could determine its own criteria for which patients would receive TARGIT boost rather than TARGIT APBI.

Sperk et al. analyzed recurrences in the Mannheim cohort of TARGIT-A patients.^15^ Among 54 TARGIT-A patients, 37 % were converted from TARGIT APBI to TARGIT Boost because of risk factors Sperk et al. chose for conversion, which included larger tumors (>2 cm) with narrower margins (<10 mm). With a median follow-up of 40 months, they report no recurrences in the 34 patients who received TARGIT APBI. Notably, 80 % of their patients also received adjuvant endocrine therapy, which could delay the appearance of recurrences. Nevertheless, if these good results are sustained with longer follow-up and can be replicated by other centers, it is possible that T1 tumors and wide excision surgery with adjuvant endocrine therapy could form a basis for “risk-adapted” TARGIT treatment. The variability of standards from center to center in the TARGIT-A Trial makes it more difficult to identify which cohort of women might benefit from this treatment strategy.

Prepathology women meeting the general TARGIT-A inclusion criteria appear to be the best candidates. However, at least 20 % of women who receive TARGIT treatment will also require 5 weeks of EBRT. Because the TARGIT-A study allowed treatment centers to determine the risk factors that required an additional 5 weeks of treatment, the Trial provides no guidance to new adopters as to when it is appropriate to add additional treatment.

The volume of tissue irradiated with the TARGIT technique is of concern because dose decreases rapidly with distance from the applicator surface. Even assuming favorable radiobiological equivalence, only tissue within a few mm of the applicator surface receives as much as a 50-Gy EBRT equivalent dose.

In the Milan III Trial, quandrantectomy alone was insufficient to achieve local control in early-stage breast cancer, even though 20 mm of tissue beyond the tumor was excised in all directions.^16^ At 10 years, local recurrence rates in patients receiving quandrantectomy alone vs those also receiving quadrantectomy plus 5 weeks of EBRT was 23.5 versus 5.8 %, respectively, with the difference less in older patients.

A multicenter randomized trial in women older than 55 years compared wide excision surgery alone (1 cm clear margins) with wide excision surgery plus 5 weeks of EBRT with an EBRT boost.^17^ Almost all patients received adjuvant hormonal therapy. With a median follow-up of 9 years, the local recurrence rates were 4.4 % for excision alone versus 3.4 % for excision plus radiation therapy, *p* = NS.

In TARGIT-A, the combination of surgical excision and effective radiation treatment depth is less than in Milan III, and in some cases, even less than 10 mm total. At 29 months median follow-up, the TARGIT-A postpathology (all of whom received a single-dose treatment in a second procedure) had local recurrence rates of 5.7 %, whereas prepathology patients (21 % of whom also received 5 weeks of WBI) had local recurrence rates of 2.1 %.

Fewer deaths were observed in the TARGIT arm than the EBRT arm, 37 versus 51, *p* = 0.008 (Table 3). The TARGIT authors assert that TARGIT treatment, while resulting in higher local recurrence rates, leads to an overall improvement in survival due to fewer non-breast cancer deaths. This conclusion is one of the main findings in the TARGIT-A update publication.^7^ The authors recommend that clinicians advise patients that while TARGIT bears a higher risk of local recurrence, TARGIT may decrease overall mortality by 2.3 %.

**Table 3:** Causes of death as reported in TARGIT-A update

	All deaths		Breast deaths and cardiac deaths, only					
	TARGIT	EBRT	TARGIT	EBRT	Targit prepath	EBRT prepath	Targit postpath	EBRT postpath
Breast cancer	20	16	20 (2.6 %)	16 (1.9 %)	17 (3.3 %)	15 (2.7 %)	3 (1.2 %)	1 (0.5 %)
Other cancers	8	16	*p* = 0.56		*p* = 0.72		*p* = 0.35	
Cardiac death	2	8	2	8	NS	NS	NS	NS
Strokes	0	2						
Ischemic bowel	0	1						
Other deaths	7	8						
Total	37	51	22^a^	24^a^				

Adapted from Table 2, Lancet^7^ w/Breast Cancer Deaths added

*NS* not stated

^a^Death due to breast cancer and cardiac events together

Harness et al. and Yarnold et al. argue that it is impossible for the 12-year-old Targit study, with a median follow-up of 29 months, to impact other cancer deaths, since the latency period for inducing non-breast cancers from breast treatment is known to be 15–20 years.^18^^,^ 19 Furthermore, deaths from stroke and ischemic bowel disease cannot be attributed to breast irradiation. If you include only cardiac and breast cancer deaths, the difference between treatment arms is only two patients. Significance in only achieved by including deaths that are unrelated to radiation treatment.

Mackenzie et al., Yarnold et al., and Harness et al. argue that Vaidya et al.’s assertion (fewer cardiac deaths from TARGIT) is inconsistent with the Darby study, the very study cited 1 in support of this claim.^1^^,^ 7 18 ^–^ 22 Mackenzie et al. suggest differences in baseline cardiac risk factors in the study groups are the most likely explanation for finding more cardiac deaths in the EBRT arm.^20^ Vaidya et al. concede that cardiovascular assessment was not recorded prior to study entry, but speculates that IORT of the tumor bed might have systemic beneficial effects that contribute to reduction in non-breast cancer mortality.^22^ However, this theory was not confirmed in the more mature ELIOT study, which showed no differences in non-breast cancers and overall survival, even out to 10 years of follow-up.^23^

## TARGIT-A Conclusions

The TARGIT-A trial, like the ELIOT Trial, included patients that today would not be considered the best choice for APBI. TARGIT-A has contributed to our understanding of whether a 1-day treatment may be possible, this time using 50-kV X-rays. With 29 months of median follow-up, the TARGIT Data are still immature and risk-adapted IORT with 50-kV X-rays is still too early in follow-up to select the subset of women whose local control will be within their noninferiority criteria margin of 2.5 %. Prepathology patients who meet the TARGIT-A inclusion criteria appear to be the best candidates and, at this point, show encouraging results. Until the data are more mature, 50-kV patients should be treated under strict institutional protocols. When long-term results are available, it is likely there will be a higher overall recurrence rate for TARGIT when compared with EBRT, but, as with ELIOT, we may be able to select subgroups of favorable patients where this difference is small and acceptable. How much additional risk of local recurrence is acceptable will vary with patients and the situation in which they find themselves.
